# Turning the screw: assessing the impact of full power-assisted versus manual pedicle screw insertion in paediatric spine surgery

**DOI:** 10.1007/s43390-025-01138-4

**Published:** 2025-07-05

**Authors:** Weronika Nocun, Neel Badhe, Vasanth Bharathidasan, Gayathri Vimal, Kalyani Parvathi Nair, Elie Najjar

**Affiliations:** 1 School of Medicine, University of Nottingham, Nottingham, UK; 2 Centre for Spinal Studies and Surgery, Queens Medical Centre, Nottingham University Hospitals National Health Service (NHS) Trust, Nottingham, UK; 3 School of Clinical Medicine, University of Cambridge, Cambridge, UK; 4 Amrita Institute of Medical Science and Research, Kochi, India

**Keywords:** Scoliosis, Pedicle screw placement, Manual pedicle screw insertion, Power-assisted pedicle screw insertion, Surgeon injury

## Abstract

**Background:**

Long-level pedicle screw placement involves repetitive, forceful rotational movements that increase the risk of musculoskeletal pain and disorders in spine surgeons. Full power-assisted pedicle screw insertion may mitigate these risks, but its efficacy and safety compared to manual techniques remain underexplored. Understanding these differences is crucial for optimizing surgical practices and improving outcomes.

**Objective:**

To systematically evaluate the outcomes of full power-assisted versus manual pedicle screw insertion in paediatric spine surgery, focusing on operative time and complications.

**Methods:**

A systematic review of English-language literature published up to September 2024 was conducted using the search criteria ("Pedicle Screws"[Mesh]) and (power), adhering to PRISMA guidelines. Studies comparing full power-assisted and manual pedicle screw insertion were included.

**Results:**

Of 2,559 patients, 1,715 underwent full power-assisted insertion (65.7% female, mean age 14.3 years, mean follow-up 20.8 months), and 844 underwent manual insertion (67.9% female, mean age 14.5 years, mean follow-up 26.9 months). Operative times were comparable (248 vs. 251.4 min, *p* = 0.69), as were screw breach rates (0.49% vs. 1.42%, *p* = 0.23). However, manual insertion was associated with a significantly higher rate of other complications compared to full power-assisted techniques (0.077% vs. 0.022%, *p* = 0.03).

**Conclusion:**

Full power-assisted pedicle screw insertion is as efficient and accurate as manual techniques, with fewer complications in pedicle screw placement surgery. These findings support the use of power assistance to enhance surgical safety. Further research should validate these results in diverse patient populations and long-term follow-up.

## Introduction

There has been an increasing prevalence of spine surgery in recent years, with studies estimating that the annual number of procedures has more than doubled in the last 20 years [6, 20]. Scoliosis surgery represents a large proportion of these cases, particularly in paediatric patients, highlighting the need to optimise treatment strategies and outcomes for these patients [16]. Scoliosis, a complex three-dimensional spinal deformity characterized by lateral curvature and vertebral rotation, affects individuals physically, emotionally, and socially [17, 26, 31]. While the majority of cases are managed conservatively, approximately 1% of patients require surgical intervention due to debilitating back pain or progressive deformity [17, 26]. Left untreated, the condition can lead to significant cosmetic and orthopaedic impairments, adversely impacting mental health and quality of life [26, 31]. Early detection and timely management are critical to mitigating these effects and improving long-term outcomes [19]. Pedicle screw fixation has become the cornerstone of scoliosis correction surgery, offering superior three-dimensional control of spinal alignment [24]. However, the manual insertion of pedicle screws involves repetitive and forceful rotational movements, contributing to musculoskeletal pain and work-related disorders in spine surgeons, with studies estimating prevalence rates as high as 52% [1, 4, 25]. To address these ergonomic challenges, full power-assisted tools have been introduced, demonstrating improvements in surgical comfort, efficiency, and control. For instance, power-assisted techniques have been shown to reduce the need for screw revisions or removals by 16.7% and decrease fluoroscopy time by 32% compared to manual methods [32].

Despite these advantages, widespread adoption of power-assisted tools remains limited. Concerns over the loss of tactile feedback during screw placement, which provides critical insights into pedicle integrity, persist [5, 18]. In addition, serious complications such as neurovascular injuries associated with power-assisted techniques have raised safety concerns [15, 27, 28, 36]. These factors contribute to ongoing debates about the balance between surgeon ergonomics and patient safety in scoliosis surgery.

This systematic review seeks to address these issues by critically evaluating the existing literature comparing manual and power-assisted pedicle screw placement techniques in scoliosis correction surgery. By analysing key metrics such as operative time, screw accuracy, and complication rates, this review aims to provide evidence-based guidance to optimise surgical decision-making and outcomes.

## Methods

### Search strategy

A systematic literature review was done using Preferred Reporting Items for Systematic Reviews and Meta Analyses (PRISMA) guidelines. The search was conducted on PubMed, Web of Science, Cochrane and Embase dating up to September 2024.

The inclusion and exclusion criteria are detailed in Table 1 below.

**Table 1:** Table illustrating the inclusion and exclusion criteria used in this study

Inclusion criteria	Exclusion criteria
Paediatric patients undergoing surgery for pedicle screw insertion by manual or power-assisted tools due to scoliosis (adolescent idiopathic, early-onset, juvenile, atypical, congenital, neuromuscular), syndromic conditions, or trauma	Studies that did not compare manual and power-assisted tools
Studies comparing manual and power-assisted tools for pedicle screw insertion	Studies that did not insert pedicle screws
Studies reporting clinical outcomes, including operative time and complications	Non-English language studies
Studies using solely the manual technique for pedicle tract preparation	Case reports or conference abstracts
Studies using solely the power-assisted tool technique for pedicle tract preparation	Studies using a combination of manual and power-assisted techniques for pedicle tract preparation
	Studies whose full-text versions were unobtainable

The PICO framework is presented in Table 2 below.

**Table 2:** Table illustrating the PICO framework for defining systematic reviews

Population	Intervention	Comparison	Outcome
Paediatric patients undergoing pedicle screw insertion due to scoliosis, syndromic conditions, or trauma	Power-assisted pedicle screw placement	Manual pedicle screw placement	Operative time and complications

The search terms used included "(Pedicle Screws"[Mesh]) or (pedicle screw) and (power)”.

**Fig. 1:**
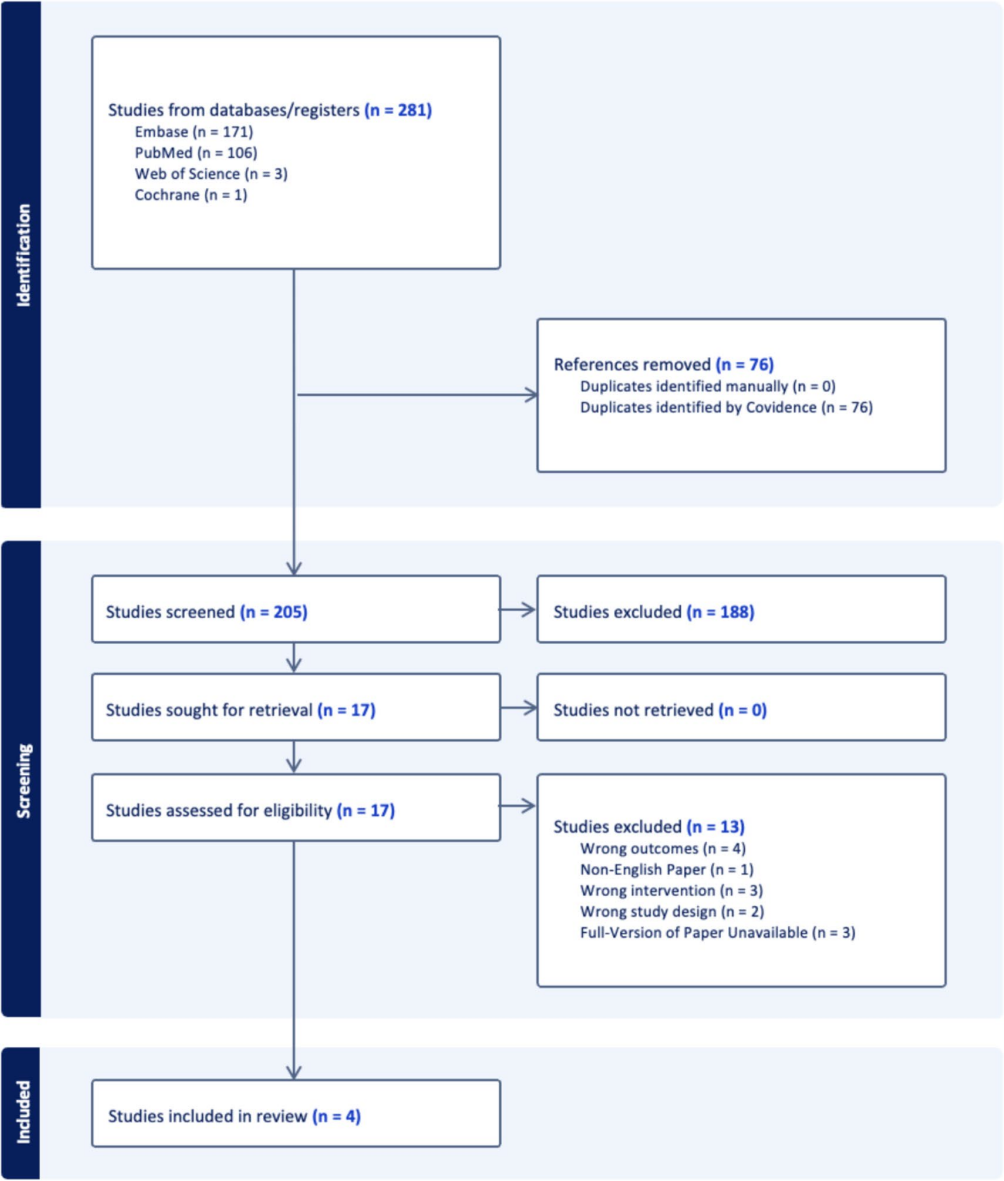
Figure illustrating the PRISMA chart for this systematic review

Duplicated articles were removed prior to screening by Covidence [8]. Two researchers independently screened the remaining studies by title and abstract, using the inclusion and exclusion criteria. The articles that remained underwent a comprehensive full-text screening by two authors independently. Any disagreements were resolved through discussion with a third researcher. The PRISMA chart can be seen in Fig. 1 below.

### Data collection

Multiple data points were collected during the extraction process, including the title, year, source, level of evidence, study design, country of study conduction, sample size, age, gender, operative time, screw density, instrumented levels, number of screws per case, follow-up, and complications. Data collection was performed by two authors.

### Risk of bias assessment

According to the MINORS criteria, the items were scored 0 if not mentioned, 1 when the item was reported but deemed inadequate, and 2 when the item was reported and deemed adequate. The total score was 16 for non-comparative studies and 24 for comparative studies [34].

All studies included are comparative studies. The total percentage was calculated for each study and was interpreted as follows [29].

- < 50%: high risk of bias;
- 50–80%: moderate risk of bias;
- > 80%: low risk of bias.

All studies included in this review fall into the category of moderate risk of bias (Table 3).

**Table 3:**
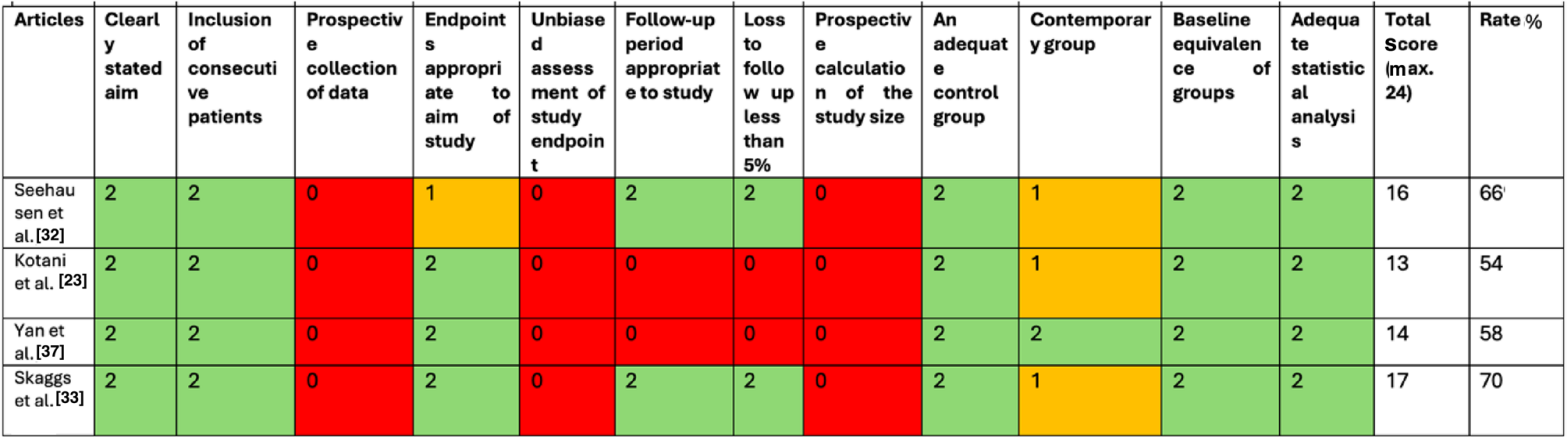
Table illustrating the MINORS criteria for defining risk of bias. Red cells signify this criterion was not reported or inadequately assessed, orange cells signify the criterion was reported but is unclear, and green cells signify the criterion is adequately reported

### Statistical analysis

Demographic statistics were calculated using GraphPad Prism version 10.3.1. Unpaired t tests were performed for continuous variables, and Chi-square tests were used for categorical variables. Meta-analysis of pooled data was performed using RevMan 5 using fixed or random-effects models. Mean difference was used for continuous variables (operative time) whereas risk ratios were used for categorical variables (screw breach and complication rate). Heterogeneity was quantified using the *I*^2^ statistic: fixed-effect models were used when heterogeneity was low (*I*^2^ < 50%) and random-effects models were used when heterogeneity was high (*I*^2^ > 50%).

## Results

### Overview of included studies

This systematic review analysed four studies selected from an initial pool of 281 identified articles [23, 32, 33, 37]. The majority of studies were Level 3 evidence, with one study classified as Level 2 evidence. The level of evidence was determined using the Oxford Centre for Evidence-Based Medicine (CEBM) guidelines [30]. Details of the included studies are summarized in Table 4.

**Table 4:** Table detailing the studies included, alongside their design, country of origin, and level of evidence

Number	ID (last name, year of publication)	Design	Country	Level of evidence
1	Seehausen et al. [32]	Retrospective cohort study	USA	3
2	Kotani et al. [23]	Retrospective cohort study	Japan	3
3	Yan et al. [37]	Prospective randomised controlled trial	China	2
4	Skaggs et al. [33]	Retrospective cohort study	USA	3

The studies used either free-hand techniques with fluoroscopy (three studies) or O-arm navigation (one study). Navigation, as used in Kotani et al., reduced fluoroscopy time compared to free-hand methods.

A total of 2,559 paediatric patients were included in the analysis, with 1,715 patients undergoing full power-assisted pedicle screw insertion and 844 patients treated using the manual technique. The full power-assisted group utilized power tools for both canal preparation and screw placement, while the manual group relied exclusively on manual techniques, as described by Seehausen et al. [32].

The indication for pedicle screw placement varied between each study, as can be seen in Table 5.

**Table 5:** Table detailing the indications for paediatric spine surgery

References	Intervention type (N)	Indication (n of patients)							Total number of patients
		Adolescent idiopathic scoliosis	Other idiopathic scoliosis (idiopathic subset included early-onset, juvenile, adolescent, and atypical idiopathic scoliosis)	Congenital scoliosis	Neuromuscular scoliosis	Syndromic conditions	Spondylolysis/spondylolisthesis	Trauma	
Seehausen et al. [32]	Manual (159)		87	24	22	23	2	1	
	Power (283)		146	40	50	37	7	3	442
Kotani et al. [23]	Manual (30)	30							
	Power (30)	30							60
Yan et al. [37]	Manual (70)	70							
	Power (35)	35							105
Skaggs et al. [33]	Manual (585)	381		30	100	45	7	22	
	Power (1367)	753		110	349	81	47	27	1952
Totals		1299	233	204	521	186	63	53	2559

### Full power-assisted group and manual group

In the full power-assisted group, 65.7% of patients were female, with a mean age of 14.3 years. The mean number of instrumented levels per case was 11.0, with 10.3 levels containing screws. The average screw density was 1.6, and the mean number of screws per case was 16.3. The average operative time for this group was 246 min, with a mean follow-up period of 20.8 months. The average fluoroscopy time was 6.9 min, and the estimated blood loss was 387.5 mL. Among 27,857 screws inserted, there were 137 breaches (0.49%) and 6 other complications (0.022%), including screw failure, neurological compromise, and visceral or vascular injury.

Of the 844 patients in the manual group, 67.9% were female, with a mean age of 14.5 years. The mean number of instrumented levels per case was 10.9, with 9.5 levels containing screws. The average screw density was 1.6, and the mean number of screws per case was 15.3. The average operative time was 249 min, with a mean follow-up period of 26.9 months. The average fluoroscopy time was 8.75 min, and the estimated blood loss was 377.5 mL. Among 12,926 screws inserted, there were 184 breaches (1.42%) and 10 other complications (0.077%), including screw failure and neurological compromise.

### Meta-analysis

**Fig. 2:**
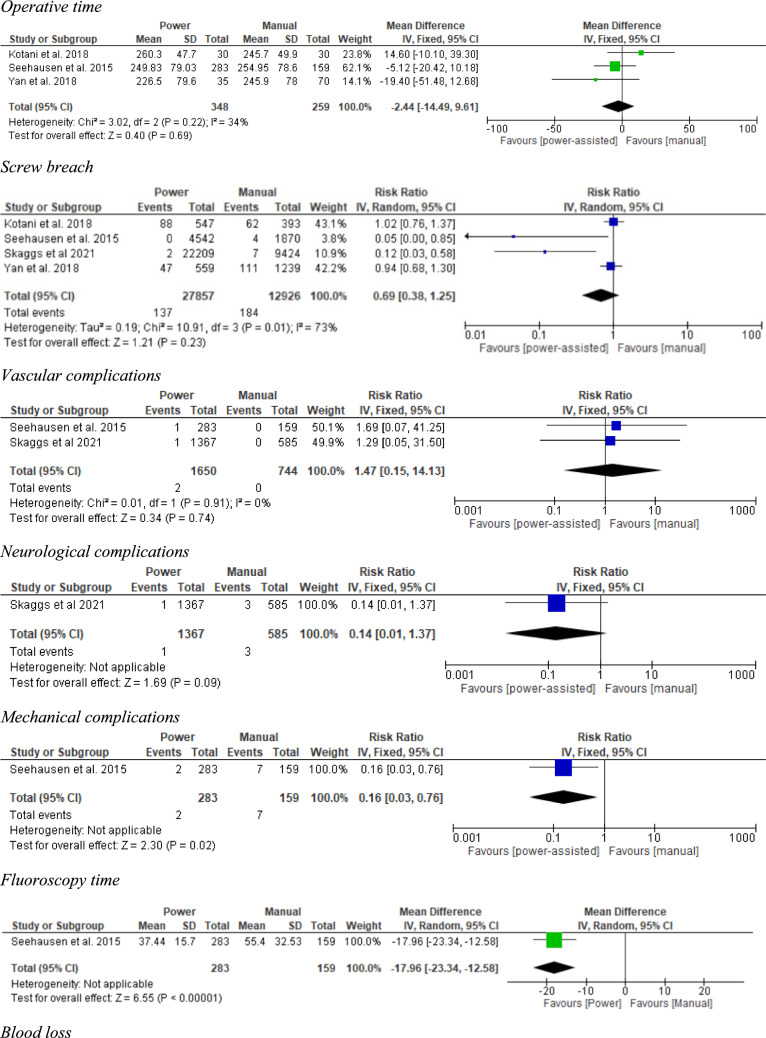

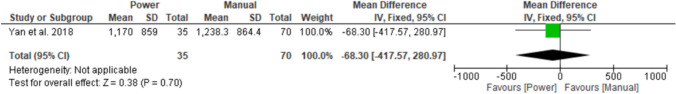
Figure detailing the operative time, screw breaches, and complications for patients with AIS

A meta-analysis compared the outcomes of full power-assisted and manual pedicle screw insertion techniques (Fig. 2):

**Operative time:** No significant difference was observed between the power and manual technique (248.4 vs 251.4 min respectively; mean difference: − 2.44; 95% CI: [− 14.49, 9.61]; *p* = 0.69).

**Screw breaches:** The rate of screw breaches showed no significant difference between the power and manual groups (0.49% vs. 1.42%; RR: 0.69; 95% CI: [0.38, 1.25]; *p* = 0.23).

**Vascular complications:** There were two vascular complications, which both occurred in the power group (0.10% vs 0.00%; RR 1.47; 95% CI: [0.15, 14.13]; *p* = 0.74).

**Neurological complications:** Only one study reported neurological complications, with one complication occurring in the power group, and three occurring in the manual group (0.07% vs. 0.51%; RR 0.14; 95% CI: [0.01, 1.37]; *p* = 0.09).

**Mechanical complications:** Similarly, only one study reported mechanical complications. Patients treated with power instruments had a significantly lower risk of mechanical complications when compared to those in the manual group (0.71% vs. 4.40%; RR 0.16; 95% CI: [0.03, 0.76]; *p* = 0.02).

**Fluoroscopy time:** Seehausen et al. reported fluoroscopy time and found that the full power-assisted group had a shorter average fluoroscopy time compared to the manual group (37.85 vs. 55.51 s; mean difference − 17.96; 95% CI: [− 23.34, − 12.58], *p* < 0.001) [32].

**Blood loss:** Yan et al. found that the average blood loss was slightly lower in the full power-assisted group compared to the manual group, but this difference was not statistically significant (1238.3 mL vs. 1170.0 mL; mean difference − 68.30; 95% CI: [− 417.57, 280.97], *p* = 0.70) [37].

These findings highlight the comparable efficacy of both techniques in terms of operative time and screw breach rates, with power-assisted methods offering potential ergonomic advantages, including reduced fluoroscopy time and slightly lower blood loss, as well as significantly lower complication rates.

## Discussion

This systematic review included four studies comparing full power-assisted pedicle screw insertion (1,715 patients) to manual insertion (844 patients). The results indicate that the power-assisted technique offers a significantly lower rate of complications, with comparable operative times and screw breach rates.

### Operative time, fluoroscopy use, and blood loss

While the difference in operative time between power and manual techniques was not statistically significant (248.4 vs. 251.4 min respectively, *p* = 0.69), full power-assisted tools demonstrated a 32% reduction in fluoroscopy [32]. The power-assisted group averaged 37.85 s of fluoroscopy time compared to 55.51 s in the manual group (*p* < 0.001). The reduction in fluoroscopy time may be attributed to enhanced control and accuracy with power tools, decreasing the need for prolonged imaging. A shorter fluoroscopy time is a notable ergonomic and safety advantage, minimizing radiation exposure for both the surgical team and the patient [3, 9, 10, 14, 35]. These findings are supported by Kojima et al. who used a hybrid technique and found that using power tools to insert pedicle screws is on average 16.9 s faster than inserting them manually [21].

Blood loss was comparable between the two groups, with the power-assisted group averaging 1238.3 mL compared to 1170.0 mL in the manual group (*p* = 0.70). The small difference is unlikely to have clinical significance but contrasts findings from some studies reporting slight reductions in blood loss with power-assisted techniques [12]. These results highlight that while power-assisted tools may offer ergonomic and safety benefits, their impact on operative blood loss remains minimal.

### Complications

Malpositioned screws are reported as one of the most common complications in surgeries involving pedicle screw placement [15]. Pedicle screw misplacement can lead to severe and sometimes irreversible consequences, including neurovascular injury, dural laceration, pedicle fractures, screw loosening, and pseudarthrosis [27].This review found that manual pedicle screw insertion resulted in a significantly higher rate of mechanical complications compared to power-assisted techniques (*p* = 0.02). The higher rate of complications in the free-hand technique are multifactorial and may be due to increased surgeon fatigue and strain as well as inconsistent screw trajectories due to increased screw “wobble” [12, 33]. Interestingly, Faldini et al. suggest that the accuracy of pedicle screw placement may be more user-dependent as opposed to technique-dependent [12]. While the rate of screw breaches in our review was not statistically different (0.49% vs. 1.42%, *p* = 0.23), the nature and frequency of complications such as screw failure, vascular injury, visceral injury, neurological compromise, and screw migration varied between the groups.

#### Power-assisted group

The full power-assisted group had a total of six patients having complications among 27,857 screws (0.022%), including:

- **Hemopneumothorax:** Reported in one case where a misplaced screw caused thoracic injury.
- **Screw failure:** Two instances of mechanical failure or malposition that required revision surgery.
- **Neurological compromise:** Two patients with minor deficits were reported but resolved with revision.
- **Visceral or vascular injury:** Limited cases, including one iliac vein injury during pedicle tract preparation, which was resolved surgically.

Yan et al. reported no vascular, neurological, or visceral complications in their study, but 11% of screws were malpositioned, showing an overall accuracy of 89% [37]. Furthermore, whilst not explicitly listed as complications, there is concern about an increased risk of potential dural tears and vascular injury in power-assisted instruments, which may arise from the use of cutting blades [32, 37].

#### Manual group

In the manual group, complications were more frequent, with 10 patients experiencing events among 12,926 screws inserted (0.077%), including:

- **Neurological deficits:** Three patients experienced changes to motor-evoked potentials, requiring screw removal.
- **Mechanical failures:** Seven revisions occurred due to symptomatic lateral or medial breaches, or screw migration after placement.

Notably, three patients in the power-assisted group required revision surgery for asymptomatic lateral breaches, medial breaches post-trauma, or symptomatic conditions like spinal headaches. Revision surgeries for symptomatic and asymptomatic breaches post pedicle screw placement is a finding supported by the literature [11]. Manual screw insertion more commonly resulted in symptomatic breaches requiring surgical correction [32, 33].

### Comparison across studies

Skaggs et al. and Seehausen et al. demonstrated that the manual group had higher revision rates and a greater frequency of complications, including mechanical failure and neurological alterations [32, 33]. In contrast, more visceral and vascular injuries occurred in the power-assisted cohort [32, 33]. These findings suggest that while complications occurred in both groups, their severity, impact, and nature differed between the two groups.

### Power-assisted benefits

Yan et al. reported comparable screw accuracy between the power-assisted (89.0%) and manual group (87.6%), with no vascular or neurological complications in either cohort [37]. The ergonomic advantages of power tools, including reduced physical strain on surgeons and shorter fluoroscopy times, are complemented by the technique’s potential to enhance surgical accuracy and reduce complications.

### Surgeon ergonomics and safety

Spine surgeons face a high prevalence of musculoskeletal (MSK) injuries due to repetitive motions and long operative hours, with reported increases in neck pain, radiculopathy, and upper limb disorders [2, 4, 13]. Studies conducted on spine deformity correction surgeons report a nearly 100-fold increase in the prevalence of neck pain with radiculopathy and a fivefold increase in the prevalence of lumbar disc herniation with radiculopathy, when compared to the general population [4]. Increased incidence of carpal tunnel syndrome has also been reported [4]. Power tools reduce physical strain, as evidenced by Claeson et al., who found lower muscle exertion with power tools compared to manual instruments [7]. In addition, the reduced fluoroscopy time associated with power-assisted techniques minimizes radiation exposure, further protecting both surgeons and patients [3, 9, 10, 14, 35].

### Hybrid techniques

Hybrid techniques, which combine manual canal preparation with power-assisted screw placement, have become increasingly common in pedicle screw insertion. In a retrospective analysis of 457 patients, Kolesov et al. found that this hybrid approach achieved a slightly better screw position accuracy (89.6% vs. 89.1% for manual techniques), however this difference was not statistically significant (*p* = 0.189) [22]. This study also demonstrated significant reductions in surgery time (242.1 vs. 260.8 min; *p* = 0.034) and blood loss (876.4 vs. 950.4 mL; *p* = 0.041) with the hybrid technique, without an increased risk of complications. These results are supported by the findings of Kojima et al., who compared the use of a hybrid pedicle screw insertion with traditional free-hand techniques in minimally invasive spine stabilization (MISt) procedures [21]. This study showed that the hybrid technique significantly reduced the time required to insert percutaneous pedicle screws compared to manual methods (10.5 vs. 27.4 s per screw; *p* < 0.01), while maintaining comparable screw placement accuracy. These findings highlight how the hybrid approach combines precision with efficiency, presenting an effective strategy to address both safety concerns and the physical strain on surgeons associated with prolonged manual techniques.

### Limitations

This review has several limitations. Only four studies were included in the analysis, with only one randomized controlled trial (RCT) and three retrospective cohort studies. Therefore, these findings may not be representative of the broader population. Sample sizes varied significantly between studies, which could influence weighting in pooled analyses and reduce generalizability. In addition, no multi-variate analysis was conducted to control for confounding variables such as the indications for pedicle screw placement, the use of free-hand versus navigated techniques and timing bias due to varying surgical experience with the power-assisted compared to the free-hand technique. There was also no standardization in the reporting of specific outcomes, such as fluoroscopy time or blood loss, across studies. The lack of blinding during assessment introduces a potential for bias, and none of the studies used a standardized method to assess surgeon ergonomics or MSK outcomes. Larger, multi-center RCTs with standardized outcome measures are needed to provide more robust evidence supporting the efficacy and safety of power-assisted pedicle screw placement. Lastly, due to this study focusing on the paediatric spine deformity population, further research should be conducted to assess the difference in outcomes between manual and power instruments in adult spine deformity correction surgery.

## Conclusion

The findings of this review underscore the potential of power-assisted techniques to reduce surgeon fatigue and complication rates while maintaining surgical efficiency. Adoption of these tools may mitigate risks for both patients and surgeons, promoting safer and more effective spine surgery.

## Data Availability

Not applicable.
